# Copper-containing amine oxidases contribute to terminal polyamine oxidation in peroxisomes and apoplast of *Arabidopsis thaliana*

**DOI:** 10.1186/1471-2229-13-109

**Published:** 2013-08-05

**Authors:** Joan Planas-Portell, Marta Gallart, Antonio F Tiburcio, Teresa Altabella

**Affiliations:** 1 Department of Molecular Genetics, Centre for Research in Agricultural Genomics (CRAG) (CSIC-IRTA-UAB-UB), Campus UAB, Bellaterra (Cerdanyola del Vallès), Barcelona, Spain; 2 Laboratory of Plant Physiology, Faculty of Pharmacy, University of Barcelona, Barcelona, Spain

## Abstract

**Background:**

Polyamines (PAs) are oxidatively deaminated at their primary or secondary amino-groups by copper-containing amine oxidases (CuAOs) or FAD-dependent amine oxidases (PAOs), respectively. Both enzymes have long been considered to be apoplastic proteins. However, three out of five PAO isoforms in *Arabidopsis thaliana* are localized in peroxisomes, while the other two PAOs are predicted to be cytosolic. Interestingly, most of these PAOs do not contribute to terminal PA oxidation, but instead are involved in the back-conversion pathway, producing spermidine from spermine and putrescine from spermidine, which in turn is inhibited by putrescine. This opens the question as to whether PAs are catabolized in the apoplast of Arabidopsis and if the terminal oxidation occurs in the peroxisomes. The main objective of this study was to know if these catabolic processes are mediated by CuAOs.

**Results:**

*A. thaliana* contains ten genes annotated as CuAOs, but only one (*ATAO1*) has been characterized at the protein level. Reported herein is the characterization of three genes encoding putative Arabidopsis CuAOs (*AtCuAO1*, *AtCuAO2* and *AtCuAO3*). These genes encode functional CuAOs that use putrescine and spermidine as substrates. AtCuAO1, like ATAO1, is an extracellular protein, while AtCuAO2 and AtCuAO3 are localized in peroxisomes. The three genes present a different expression profile in response to exogenous treatments, such as application of abcisic acid, methyl jasmonate, salycilic acid, flagellin 22 and wounding.

**Conclusions:**

PA catabolism in the Arabidopsis apoplast is mediated predominantly by CuAOs, while in peroxisomes the co-localization of CuAO-dependent terminal catabolism with PAO-back-conversion machineries might contribute to modulating putrescine-mediated inhibition of the back-conversion, suggesting the occurrence of a tight coordination between both catabolic pathways. The expression profile of *AtCuAO1-3* in response to different exogenous treatments, together with the different localization of the corresponding proteins, provides evidence for the functional diversification of Arabidopsis CuAO proteins.

## Background

The polyamines (PAs) putrescine (Put), spermidine (Spd), and spermine (Spm) are low-molecular-weight organic cations found in a wide variety of organisms. In plants, polyamines are involved in different physiological processes, such as growth, development, and response to abiotic and biotic stresses [1-4].

PAs are oxidatively deaminated by amine oxidases (AOs), including those that are FAD-dependent (PAO, EC 1.5.3.6) and copper-containing (CuAO, EC 1.4.3.6) [5]. PAOs catalyse the oxidative deamination of Spm, Spd and/or their acetylated derivatives at the secondary amino group [6]. The chemical identity of PAO reaction products depends on the enzyme source and reflects the mode of substrate oxidation. Thus, in monocotyledonous plants, PAOs catalyse the terminal catabolism of PAs oxidizing the carbon at the *endo*-side of the N^4^-nitrogen of Spd and Spm to produce 4-aminobutanal and N-(3-aminopropyl)-4-aminobutanal, respectively, in addition to 1,3-diaminopropane (DAP) and H_2_O_2_[6,7]. In contrast, in the dicotyledonous model plant *Arabidopsis thaliana*, PAOs, namely AtPAO1, AtPAO2, AtPAO3 and AtPAO4, oxidize the carbon at the *exo*-side of the N^4^-nitrogen of Spd and Spm, giving rise to a PA back-conversion pathway, with the production of Spd from Spm and Put from Spd, in addition to 3-aminopropionaldehyde and H_2_O_2_[8-12].

CuAOs catalyse the oxidation of PAs at the primary amino group, giving the corresponding aminoaldehydes, with concomitant production of H_2_O_2_ and NH_3_, but in contrast to PAOs, they are unable to produce the oxidative splitting of PAs by reaction with secondary and tertiary amino groups [13,14]. CuAOs are homodimeric proteins, consisting of 70–90 kD subunits, each containing a copper ion and a 2,4,5-trihydroxyphenylalanine quinone cofactor (TPQ) generated by a post-translational autocatalytic modification of a tyrosine residue in the active site [15]. Although the overall primary sequence identity of CuAO from different sources is usually not high (<25%) [14], thirty-three amino acid residues near the catalytic site are fully conserved in most of them [16,17].

Plant AOs contribute to important physiological processes, including plant growth and development, response to abiotic stresses such as drought, salinity, and heat, and defence responses against pathogens [6,7]. These effects are mediated not only through the regulation of cellular PA levels, but mainly by the AO reaction products: aminoaldehydes, GABA (γ-aminobutiric acid) and, markedly, H_2_O_2_[1,2,6,7,18].

CuAOs and PAOs, abundant in the apoplast of Fabaceae and Gramineae, respectively, have long been considered characteristic of these two plant families, leaving PA catabolism uncovered in other plant families and underestimating their potential contribution in other cellular compartments. More recently, a number of CuAOs and PAOs have been detected in several taxa and the occurrence of AOs in intracellular compartments has been demonstrated [6,7]. Indeed, a peroxisomal localization has been reported for three of the five PAO isoforms present in *A. thaliana* (AtPAO2, AtPAO3 and AtPAO4), while the other two (AtPAO1 and AtPAO5) are predicted to be cytosolic proteins [12]. Moreover, as mentioned above, the AtPAOs oxidize PAs through the back-conversion pathway [12]. Questions then arise as to whether some AOs, localized in the same subcellular compartment as the Arabidopsis PAOs, are able to catalyse the terminal oxidation of PAs and how PAs are oxidized in the apoplast of this model plant. CuAOs occur at high levels in dicots and are the most abundant soluble protein detected in the extracellular fluids of several Fabaceae (pea, chickpea, lentil and soybean) [19]. The genome of *A. thaliana* contains ten genes annotated as copper-binding amine oxidases, but only one (*ATAO1*) has been characterized at the protein level, and it has been predicted that ATAO1 is an extracellular protein [17].

In this work, we have characterized three of the other nine genes encoding putative CuAOs in Arabidopsis (*At1g62810*, *At1g31710* and *At2g42490*). Our results indicate that these genes encode functional CuAOs with differential cellular localizations in the apoplast and peroxisomes. The expression profile of these genes in different tissues and in response to several exogenous stimuli point to a potential specification in their biological roles. Finally, considering our results and previous reports, we propose a model by which PA catabolism can be accomplished in Arabidopsis.

## Results

### *AtCuAO1*, *AtCuAO2* and *AtCuAO3* sequence comparison

**Figure 1:**
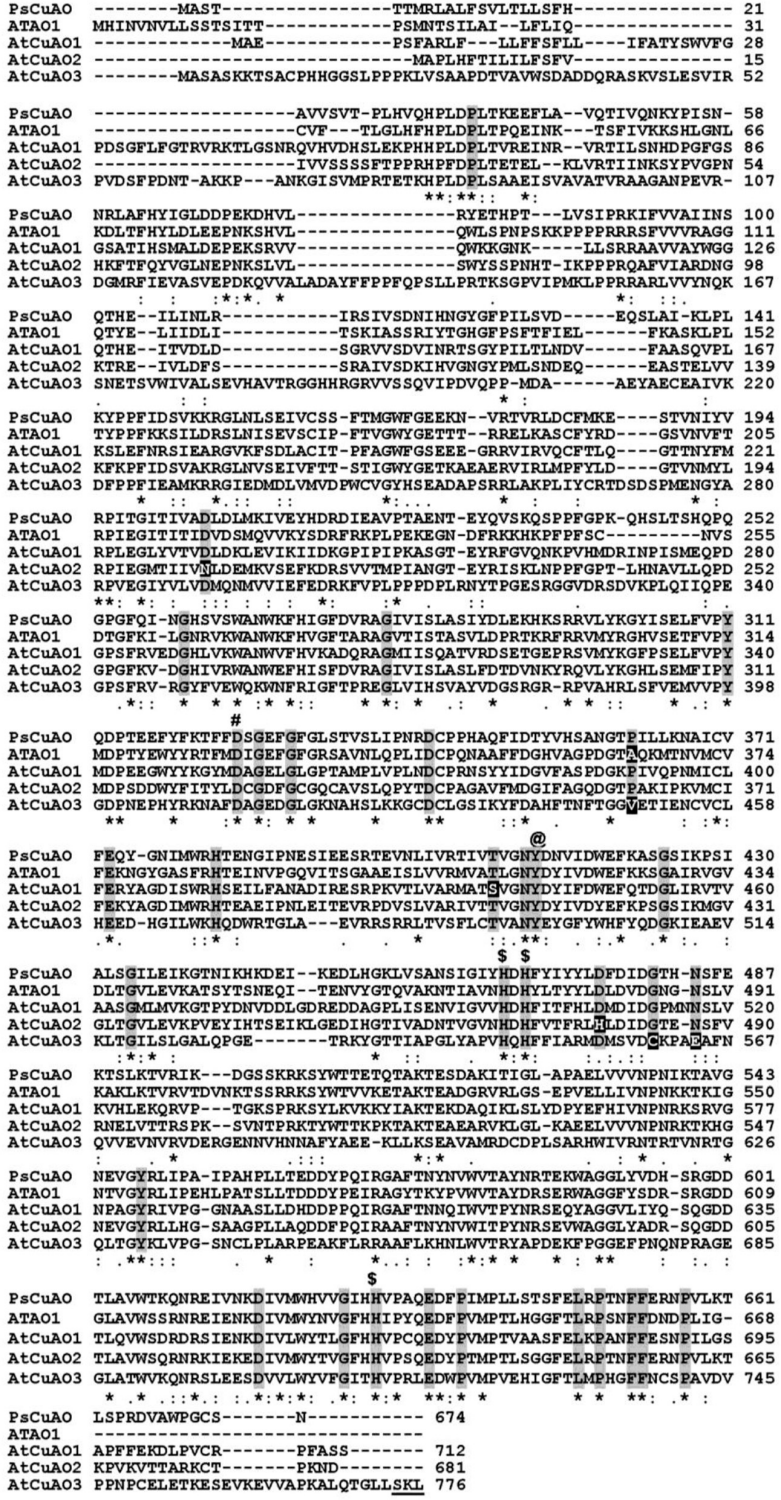
**Amino acid sequence alignment of AtCuAO1, AtCuAO2 and AtCuAO3 with other plant CuAOs.** Organism symbols (GenBank accession numbers): AtCuAO1 (NM_104959), AtCuAO2 (NM_102906), AtCuAO3 (AY120717) and ATAO1 (NM_117580), *Arabidopsis thaliana* CuAOs; PsCuAO (L39931), *Pissum sativum* CuAO. Identical residues are marked with asterisks. Conserved and semi-conserved substitutions are denoted by ‘:’ and ‘.’, respectively. The 33 residues totally conserved in most of the CuAOs are indicated by grey boxes. The residues not conserved in the *A. thaliana* CuAOs are inversely highlighted. The copper binding histidine residues and the tyrosine modified to TPQ are marked with $ and @, respectivelly. The aspartic acid active site base is indicated by #. The peroxisomal targeting signal of AtCuAO3 is underlined. The analysis was accomplished using the ClustalW sequence alignment.

In various plant species *CuAO* genes consist of a multigene family [16,17]. A search of the Arabidopsis genome database revealed the presence of ten genes encoding putative CuAOs (Table 1). We selected the three more highly expressed genes in the GENEVESTIGATOR database (http://www.genevestigator.com) [20] for further studies: *At1g62810* (*AtCuAO1*[21]), *At1g31710* (referred to as *AtCuAO2*) and *At2g42490* (referred to as *AtCuAO3*). The deduced amino acid sequences of these three putative Arabidopsis CuAOs were aligned with other known plant CuAOs (Figure 1). AtCuAO1 and AtCuAO2 showed a high level of sequence conservation relative to previously characterized plant CuAOs, with a lower level observed in AtCuAO3. In particular, the amino acid sequence of AtCuAO1 exhibited 60% similarity to ATAO1 and 64% to the CuAO of *Pisum sativum* (PsCuAO) [GenBank: L39931]. The amino acid sequence similarity between AtCuAO2 and ATAO1 was 65%, and 71% between AtCuAO2 and PsCuAO. However, the sequence similarity of AtCuAO3 with ATAO1 and PsCuAO was lower, 45% and 44%, respectively*.* The amino acid sequence similarity between AtCuAO3 and the other two AtCuAOs was also low, 44% with AtCuAO2 and 43% with AtCuAO1, while AtCuAO2, exhibited 63% similarity with AtCuAO1. Amino acid residues shown to be important for the catalytic activity of plant CuAOs are apparently conserved in the three AtCuAOs studied in this work (Figure 1). ATAO1 contains 32 of the 33 amino acid residues that have been reported to be fully conserved in CuAOs from different sources [16,17], while AtCuAO1, AtCuAO2 and AtCuAO3 contain 32, 31 and 30 of these amino acids, respectively (Figure 1), including the active site tyrosine (Figure 1, Tyr441, Tyr412, Tyr495), which undergoes modification to form the TPQ cofactor [22,23], four histidines, three of which correspond to copper ligands [24,25] (Figure 1; His499, His501 and His662 for AtCuAO1; His472, His474 and His632 for AtCuAO2; His546, His548 and His712 for AtCuAO3) and the aspartic acid active site base [26] (Figure 1, Asp354, Asp325 and Asp412).

**Table 1:** Genes annotated as copper-containing amine oxidases in The Arabidopsis Information Resource (TAIR) database

**AGI**	**GenBank accession no.**	**Gene name**	**Reference**
At4g14940	NM_117580	*ATAO1*	[17]
At1g62810	NM_104959	*AtCuAO1*	[21]
At1g31710	NM_102906	*AtCuAO2*	This work
At2g42490	AY120717	*AtCuAO3*	This work
At1g31670	NM_102902		
At1g31690	NM_102904		
At3g43670	NM_114235		
At4g12270	NM_117297		
At4g12280	NM_117298		
At4g12290	NM_117299		

### Expression of *AtCuAO1*, *AtCuAO2* and *AtCuAO3* in *N. benthamiana* and characterization of the recombinant-TAP fusion protein

To ensure that *AtCuAO1*, *AtCuAO2* and *AtCuAO3* encode functional CuAO enzymes, they were transiently expressed in *N. benthamiana* as fusion proteins using the plant expression vector pC-TAPa [27]. The TAP (tandem affinity purification) tag, lying downstream of the *AtCuAO1*, *AtCuAO2* and *AtCuAO3* ORFs, facilitates antibody detection and protein purification.

*AtCuAO1*, *AtCuAO2* and *AtCuAO3* encode 712-, 681- and 776-amino acid proteins, respectively, with calculated molecular masses of 80.1, 76.7 and 86.7 kD, while the TAP has a calculated molecular mass of 34.3 kD.

**Figure 2:**
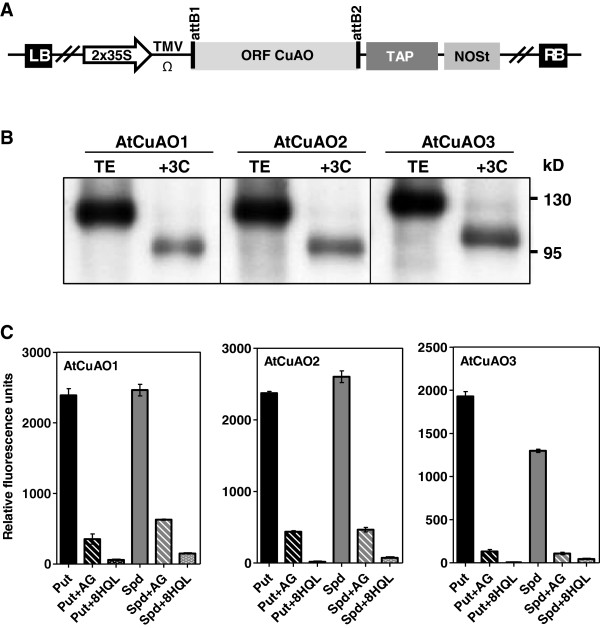
**Expression and activity of recombinant AtCuAO1, AtCuAO2 and AtCuAO3 proteins. (A)** Construct used to produce recombinant AtCuAOs in *N. benthamiana*: The PCR-amplified CuAO ORFs without the stop codon flanked by Gateway recombination cassette sequences were cloned in-frame with the TAP tag composed of: two copies of the protein A IgG binding domain, the 3C protease cleavage site, a six histidine stretch, and nine repeats of the myc epitope. The expression of TAP-tagged *AtCuAOs* is under the control of the duplicated 35S cauliflower mosaic virus promoter (2x35S), a tobacco mosaic virus translational enhancer (TMV-Ω), and a NOS terminator (NOSt). RB, T-DNA right border sequence; LB, T-DNA left border sequence. **(B)** Western blot analyses of recombinant AtCuAO proteins produced in *N. benthamiana* plants. Proteins present in total extract (TE) and in the eluate, obtained from IgG beads using 3C protease (+3C), were separated on an 8% SDS-PAGE gel and immunoblotted using the α-myc antibody. The highest MW band corresponds to the fusion protein containing the whole TAP-tag, while in the lower MW band the IgG binding domain has been cleaved. Molecular weight markers (kD) are shown on the right. **(C)** Amine oxidase activity of recombinant fusion proteins AtCuAO1-MYC9-His6 (AtCuAO1), AtCuAO2-MYC9-His6 (AtCuAO2) and AtCuAO3-MYC9-His6 (AtCuAO3) (1 μg) was determined as fluorescence corresponding to the amount of H_2_O_2_ released by oxidation of Put or Spd (1 mM), alone or pre-treated with the CuAO inhibitors aminoguanidine (AG, 0.5 mM) or 8-hydroxyquinoline (8HQL, 30 μM), using Amplex Red. Data are the means ± SE of three replicates. Bars represent standard errors.

Five days after agro-infiltration, tissues were harvested, and the soluble protein extracts were analyzed by western blot using anti-myc epitope antibodies. A single band of the expected size (114 kD, 111 kD and 121 kD) was observed for each of the three fusion proteins (AtCuAO1-TAP, AtCuAO2-TAP, AtCuAO3-TAP) (Figure 2B), which were purified by affinity chromatography using IgG-Sepharose beads and digestion with 3C protease for IgG cleavage. In the partially purified extracts the antibodies detected bands of about 101, 98 and 108 kD, which correspond to the size expected for the fusion proteins without the 2xIgG binding domain of protein A: AtCuAO1-MYC9-His6, AtCuAO2-MYC9-His6 and AtCuAO3-MYC9-His6, respectively (Figure 2B). Enzyme activity of each fusion protein was evaluated by the H_2_O_2_ production. For this, the proteins were incubated with the H_2_O_2_-sensitive Amplex red reagent, peroxidase and either Put, Spd or Spm. When any of the three proteins were incubated with Put significant oxidase activity was detected (Figure 2C), which was reduced by pre-treatment with aminoguanidine (AG), an irreversible competitive inhibitor of amine oxidases that interacts with the TPQ cofactor (Figure 2C). The oxidase activity was also reduced by pre-treatment with the copper complexing agent 8-hydroxyquinoline (8HQL), a non-competitive inhibitor of CuAOs (Figure 2C). Spd was also a good substrate for the three proteins, and the enzyme activity was again inhibited by AG and 8HQL (Figure 2C). However, none of the three fusion proteins recognised Spm as a substrate. These results suggest that AtCuAO1, AtCuAO2 and AtCuAO3 encode functional CuAOs able to oxidize Put and Spd, but not Spm, with the subsequent release of H_2_O_2_.

### Subcellular localization of AtCuAO1, AtCuAO2 and AtCuAO3

**Figure 3:**
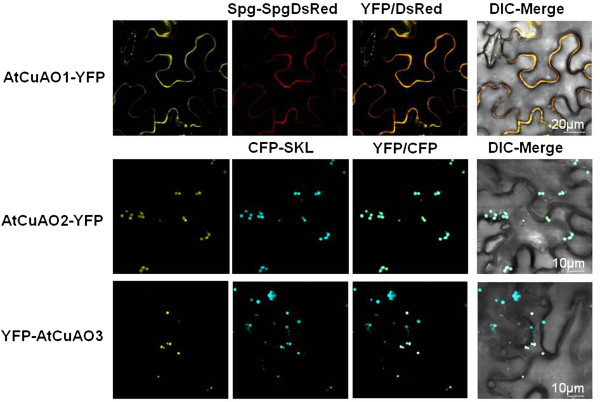
**Subcellular localization of YFP-tagged AtCuAO1, AtCuAO2 and AtCuAO3 proteins.** Confocal microscopic images showing the fluorescence localization in agroinfiltrated *N. benthamiana* epidermal leaves transiently co-expressing AtCuAO1-YFP **(A)** with the extracellular protein marker Spg-DsRed **(B)** or AtCuAO2-YFP **(E)** or YFP-AtCuAO3 **(I)** with the peroxisome protein marker CFP-SKL **(F**, **J)**. Co-localization evaluation of YFP-tagged AtCuAO proteins with the extracellular **(C** and **D)** and peroxisome **(G**, **H**, **K** and **L)** protein markers. Differential interference contrast (DIC) images showing the morphology of transformed cells and merging between the protein of interest, fused to YFP, and the marker **(D**, **H** and **L)**. The data presented are from a single representative experiment that was repeated at least three with similar results. Data were obtained using a 40x water immersion objective.

AtCuAO3 contains at its C-terminus (amino acids 774–776, Figure 1) the canonical SKL tripeptide, involved in the protein targeting to the peroxisome matrix. In fact, the peroxisomal association of this protein has been demonstrated previously by proteome data and a subcellular targeting study of YFP fusion protein [28,29]. The subcellular localization of this protein was confirmed experimentally by transiently expressing an N-terminal fusion of AtCuAO3 to the YFP in agroinfiltrated *N. benthamiana* leaf cells (Figure 3I-L). *In silico* protein localization prediction using PSORT, the only program specifically able to predict peroxisomal targeting, failed to identify any of the other nine Arabidopsis CuAOs as putative peroxisomal proteins. For AtCuAO2, the SUBA database (The Subcellular Location of Proteins in Arabidopsis Database) [30] returned a wide range of possible localizations, including extracellular, plastids, endoplasmic reticulum and cytosol, while for AtCuAO1 the possible localizations were: apoplast, vacuole, endoplasmic reticulum or mitochondria. In order to determine experimentally the subcellular localization of these two AtCuAO isoforms, YFP was fused in-frame to the C-terminus of AtCuAO1 and AtCuAO2. AtCuAO1-YFP and AtCuAO2-YFP were then transiently expressed by *Agrobacterium*-mediated infiltration of *N. benthamiana* leaves (Figure 3A-H). Confocal laser microscopy analysis of the transfected cells revealed that, as observed for YFP-AtCuAO3 (Figure 3I-L), the cells expressing AtCuAO2-YFP showed a punctuate fluorescence pattern (Figure 3E). The peroxisomes in the cells were labeled with blue fluorescence (Figure 3F ) due to the co-expression of the peroxisomal marker CFP-SKL [31]. As shown in Figure 3 (C, D, G and H), all punctuated yellow fluorescent signals in the cells coincided with the blue fluorescence of CFP-SKL, confirming that AtCuAO3 is a peroxisomal protein and strongly suggesting that AtCuAO2 is also localized in peroxisomes. Cells expressing AtCuAO1-YFP displayed a pattern of secretion, with fluorescence mainly located in the periphery of the cells (Figure 3A) as occurred in cells expressing Spg-DsRed (Figure 3B), used as a control of protein secretion [32]. Moreover, the yellow fluorescence of AtCuAO1-YFP colocalized with the red fluorescence of the secreted DsRed (Figure 3C and D), suggesting that AtCuAO1 is an extracellular protein.

### Spatial and temporal expression of three Arabidopsis *CuAOs*

**Figure 4:**
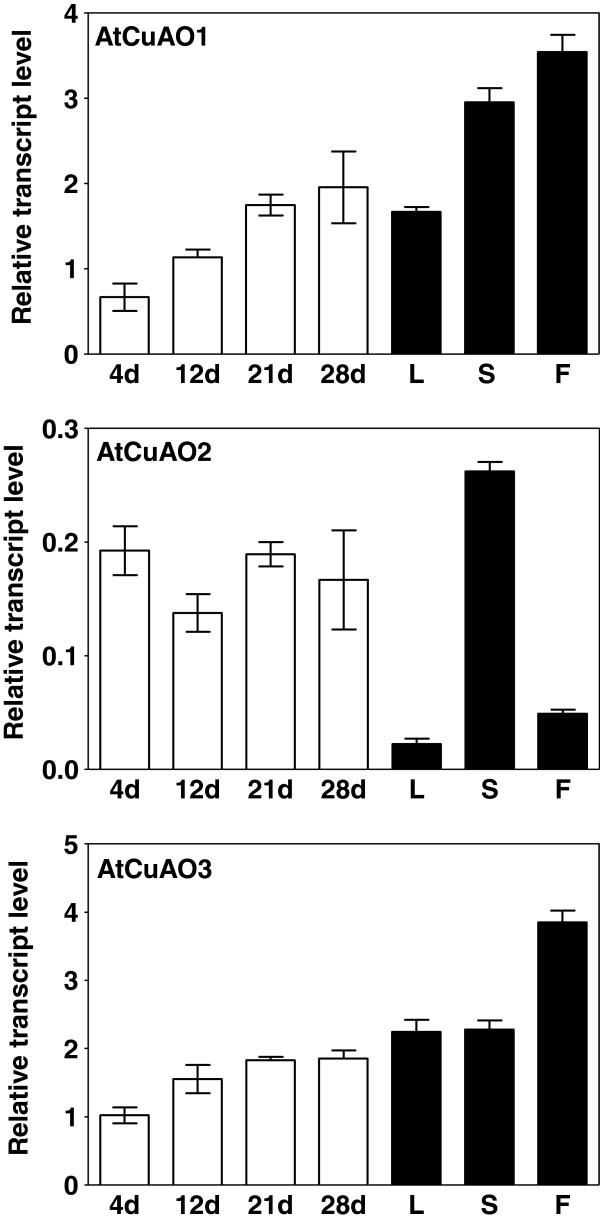
**Expression analyses of *AtCuAO *genes.** The expression of *AtCuAO1*-3 was determined in 4- to 28-day-old *A. thaliana* seedlings (white bars) and in different organs of 6-week-old plants (black bars). L, rosette leaves; S, stems; F, flowers. Transcript levels were determined by quantitative RT-PCR using gene-specific primers designed for *AtCuAO1*, *AtCuAO2* and *AtCuAO3* and are shown relative to *Actin2* in each sample. Values are the mean ± SE from three replicates. Bars represent standard errors.

To evaluate whether the different *CuAO* transcripts were spatially regulated, expression of *AtCuAO1*-*3* was examined by real-time RT-PCR using total RNA isolated from different organs of mature Arabidopsis plants (rosette leaves, stems, flowers). The expression of these genes was also determined in 4-, 12-, 21- and 28-day-old whole seedlings to know if there is some kind of regulation during plant development (Figure 4).

*AtCuAO1, AtCuAO2* and *AtCuAO3* transcripts were detected in all organs analysed. *AtCuAO1* transcripts were abundant in rosette leaves, but with the highest levels found in stems and flowers. The expression of this gene increased about 3-fold in 28-day-old seedlings when compared with those 4 days old (Figure 4). A similar expression pattern was observed for *AtCuAO3,* with transcript levels increasing during plant development and, reaching a peak in flowers of adult plants, followed by leaves and stems (Figure 4). In contrast, *AtCuAO2* transcript levels, which were abundant in stems but low in other organs, were not observed to increase during the developmental stages (Figure 4).

### Transcriptional profile of *AtCuAO1-3* in response to external stimuli

**Figure 5:**
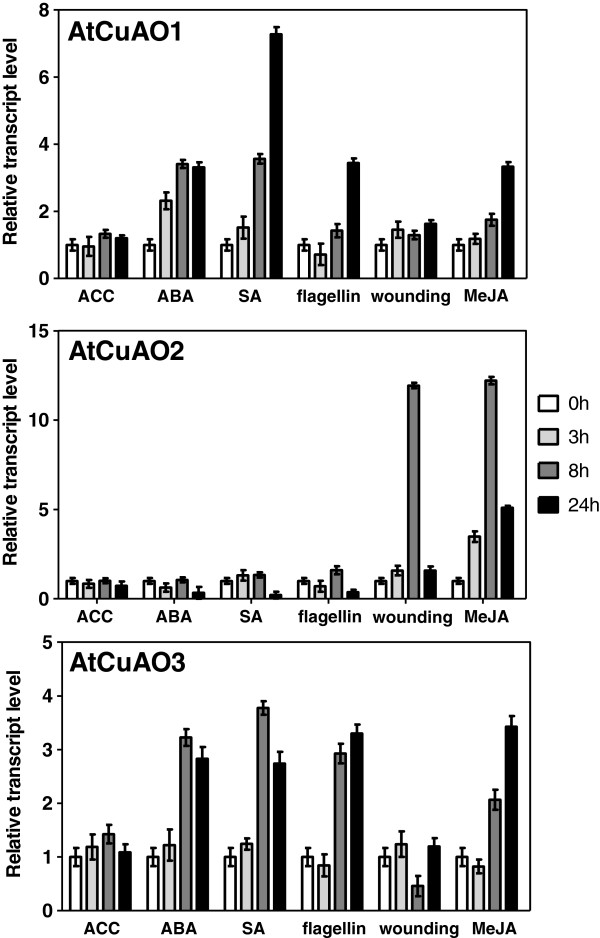
**Expression of *AtCuAO *genes in *Arabidopsis *seedlings in response to exogenous treatments.** Transcript levels of At*CuAO1, AtCuAO2* and *AtCuAO3* genes were determined by real-time RT-PCR in *Arabidopsis* seedlings exposed or not to wounding or treatment with flagellin 22, SA, MeJA, ACC or ABA after 0, 3, 8 and 24 h of treatment. Values are expressed normalized to *Actin 2* and relative to non-treated plants at each time point, which is assumed to be one. Values are the mean ± SE of three replicates. Bars represent standard errors.

CuAOs have been reported to be involved in plant response to abiotic and biotic stresses (reviewed by [6] and [7]). Thus, we decided to investigate the expression pattern of *AtCuAO1*, *AtCuAO2* and *AtCuAO3* in response to external stimuli by subjecting Arabidopsis seedlings to different treatments, including wounding, flagellin 22 (a pathogen elicitor that activates the plant basal defence), abcisic acid (ABA), salycilic acid (SA), methyl-jasmonate (MeJA), and the ethylene precursor 1-aminocyclopropane-1-carboxylic acid (ACC) (Figure 5).

The highest expression of *AtCuAO1* was observed after 24 h in SA-treated plants (about 7.5-fold higher than basal level). At this time point the *AtCuAO1* transcripts were also up-regulated in response to MeJA, flagellin and ABA, although at lower degree (about 3.5-fold). It is noteworthy that induction of this gene by ABA was already observed at 3 h post-treatment (Figure 5). *AtCuAO1* expression was not significantly affected by wounding and ACC treatment (Figure 5). *AtCuAO3* transcript accumulation was also induced by ABA, SA, flagellin and MeJA, at 8 h post-treatment (Figure 5). At 24 h the transcript levels of this gene increased in MeJA-treated plants and decreased in those exposed to ABA or SA, while they were similar to those detected after 8 h of flagellin treatment. *AtCuAO3* transcript abundance was slightly lower 8 h after wounding but returned to basal levels after 24 h, and was not significantly affected by ACC treatment (Figure 5). In contrast, while *AtCuAO2* expression was clearly induced (about 12-fold) in wounded and MeJA-treated plants at 8 h, it was not affected by any of the other applied treatments (Figure 5).

## Discussion

In Arabidopsis most studies on PA oxidation have been focused on the PAO-mediated back-conversion pathway, but little is known about the terminal oxidative deamination that leads to depletion of PA levels, which in most organisms is considered to be accomplished by CuAOs [33-35]. In this study, we have characterized three of the ten genes encoding putative CuAOs in Arabidopsis (*At1g62810, At1g31710* and *At2g42490*), referred to as *AtCuAO1*, *AtCuAO2* and *AtCuAO3*. The recombinant fusion proteins AtCuAO1-MYC9-His6, AtCuAO2-MYC9-His6 and AtCuAO3-MYC9-His6 are able to oxidize Put and Spd, but not Spm, with the corresponding delivery of H_2_O_2_, and this oxidation is inhibited by the CuAO inhibitors AG and 8HQL (Figure 2). These results demonstrated that the three studied genes encode functional CuAOs, which oxidize Put and Spd. Put has been described as the best substrate for most of the characterized CuAOs, but these enzymes are also able to oxidize Spd and Spm in plants and animals [13,14,35] All plant CuAOs thus far characterized and the intracellular animal CuAOs have a lower catalytic efficiency for Spd and Spm than Put, while animal serum CuAOs preferentially oxidize Spd and Spm [35]. Thus, Spm oxidation by some of the Arabidopsis CuAOs not yet characterized cannot be discarded.

AtCuAO2 and AtCuAO3 are localized in peroxisomes (Figure 3E-L), thus representing the first CuAOs involved in a peroxisomal catabolic pathway of PAs, since any of the animal or plant CuAOs characterized so far are localized in these organelles [34,35]. AtCuAO3 contains a peroxisome targeting signal (PTS) at its C-terminus (SKL, Figure 1) and has already been reported as a peroxisomal protein [28,29]. Although *in silico* protein localization prediction failed to identify AtCuAO2 as a putative peroxisomal protein, the localization pattern observed using in vivo targeting analysis of CuAO2-YFP fusion protein strongly suggests that it is also a peroxisomal protein (Figure 3E-H). This lack of correlation between *in silico* predictions and in vivo targeting analysis has been observed in other peroxisomal proteins and could be attributed to an insufficient knowledge of PTS variant sequences or to the action of “piggy-backing” import mechanisms that do not involve PTS sequences [29].

**Figure 6:**
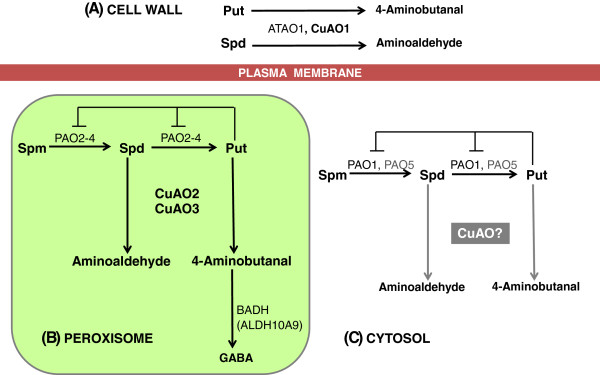
**Schematic representation of polyamine catabolic pathways in *Arabidopsis. *(A)** In the cell wall, Put and Spd can be oxidatively deaminated by CuAOs (ATAO1 and AtCuAO1) to produce the corresponding aminoaldehydes, H_2_O_2_ and NH_3_. **(B)** In peroxisomes, Spm and Spd are substrates for PAOs (PAO2-4) that catalyse the sequential back-conversion of Spm to Spd and Spd to Put, which is subjected to feedback inhibition by its end product, Put. CuAOs (AtCuAO2 and AtCuAO3) can catalyse the terminal oxidation of Put and Spd. The oxidation product of Put, 4-aminobutanal, can be a substrate for peroxisomal BADH (ALDH10A9), which catalyses its conversion to GABA. **(C)** In the cytosol, AtPAO1, and probably AtPAO5, oxidize Spm and Spd by the back-conversion pathway. Some AtCuAOs, annotated as cytosolic in TAIR, could mediate terminal PA catabolism in this cellular compartment.

Although AtCuAO3 has been identified as a peroxisomal protein, no experimental data about its function have been reported so far. The results of this work, which show that this protein, as well as AtCuAO2, oxidizes Put and Spd (Figure 2C), acquire special significance considering that none of the three AtPAOs localized in peroxisomes are able to oxidize PAs through a terminal catabolic pathway [12]. Consequently, the terminal oxidation of PAs in these organelles would be mediated by AtCuAO2 and AtCuAO3, thus avoiding the accumulation of Put, which has been described as having an inhibitory effect on the back-conversion pathway [9]. The oxidation of Put produces 4-aminobutanal, which can be further metabolized to GABA by the action of an aldehyde dehydrogenase (ADH) [36]. In Arabidopsis this process could take place in the peroxisomes mediated by a betaine aldehyde dehydrogenase (BADH) found in this organelle (ALDH10A9) [37], which possesses both betaine- and amino-aldehyde dehydrogenase activities, and can oxidize 4-aminobutanal [37] (Figure 6). These results suggest the occurrence of a tight coordination between back-conversion and terminal catabolism pathways to maintain PA homeostasis. The cooperation of the two PA catabolic enzymes is known to occur in the tobacco apoplast, where PAOs catalyse the terminal oxidation of Spd and Spm [38,39] but, to our knowledge, this is the first time that this cooperation has been reported in peroxisomes, where PAOs are involved in the PA inter-conversion reactions.

AtCuAO1 is annotated as an extracellular protein in The Arabidopsis Information Resource (TAIR). This localization was experimentally confirmed using in vivo targeting analysis of the YFP-CuAO1 fusion protein (Figure 3A-D). ATAO1, the only AtCuAO isoform characterized hitherto, has also been predicted to be an extracellular protein [17]. Since none of the characterized AtPAOs are localized in the apoplast [19], our data suggest that in the Arabidopsis cell wall the oxidative catabolism of PAs is mediated mainly (or exclusively) by CuAOs.

*AtCuAO3,* like *AtPAO3*[9], was up-regulated in response to ABA, MeJA, SA and flagellin (Figure 5). ABA-induction has also been observed in the other two genes encoding peroxisomal AtPAOs (*AtPAO2* and *AtPAO4*) [9], as well as in *ALDH10A9*[37]. These data support the idea of cooperation between the two PA catabolic pathways in Arabidopsis peroxisomes and suggest that PA catabolism in this organelle can be involved in the plant response to stress. In contrast to *AtCuAO3, AtCuAO2* was strongly induced by wounding and MeJA, but not by other stimuli (Figure 5). Therefore, both enzymes might play different roles in defence responses, AtCuAO2 being more specifically involved in the response to mechanical damage. The involvement of CuAO in wound-healing has been previously described in several plant species, such as chickpea, where *CuAO* expression was strongly induced by wounding and jasmonic acid [40], or pea, where injury induced an increase in CuAO, ADH and peroxidase activity levels, as well as in Put, Spd and GABA content [41]. In tobacco, both PAO and CuAO have been reported to participate in the wound-healing process [7,39,42]. On the other hand, the transcript level of *AtCuAO3* increased in 28-day-old seedlings compared to those 4 days old, but this was not observed in *AtCuAO2* (Figure 4), suggesting that the former gene could have a more relevant role in development. The expression profile of *AtCuAO1* (Figure 4) in growing seedlings was similar to that observed for *AtCuAO3,* suggesting that this gene could also be involved in plant development. It has been reported that the H_2_O_2_ produced by plant PA catabolism is necessary for several plant developmental processes, such as cell expansion, polar growth, gravitropism, flower development, stomata aperture and stress responses [35].

*AtCuAO1,* like *AtCuAO3,* was up-regulated by ABA, MeJA, flagellin and, to a higher degree, by SA (Figure 5), suggesting that AtCuAO1, like the peroxisomal CuAOs, could also be involved in the plant response to stress, but more studies are required to understand the role of each protein in these processes. Induction of *AtCuAO1* by ABA has been previously observed by Wimalasekera et al. [21], who showed that *cuao1* mutants present an ABA-hyposensitive phenotype and impaired H_2_O_2_ and NO production. Moreover, these mutants were less sensitive to the inhibition of root growth induced by osmotic stress [21], supporting the idea that AtCuAO1 is involved in plant response to abiotic stress. The contribution of apoplastic PA catabolism to stress defense responses has been described in other plant species using PAO-specific inhibitors and transgenic plants [34,35]. Thus, using transgenic tobacco plants overexpressing (S-PAO) or downregulating apoplastic *PAO* (A-PAO), Moschou et al. [43] demonstrated that salinity induces the exodus of Spd into the apoplast, where it is catabolized by PAO producing H_2_O_2_ that induces either tolerance responses or programmed cell death, depending on the levels of intracellular PAs. Moreover, S-PAO plants were more tolerant to specific pathogens that depend of the apoplast for their growth, like the bacteria *Pseudomonas syringae* and the oomycete *Phytophora parasitica* var. *nicotianae*. Furthermore, the high PA oxidation in the apoplast of these plants preinduces responses such as the expression of SAR-linked genes and increased cell wall-based defense, showing that PAO is a nodal point in a specific apoplast-localized plant-pathogen interaction, at time that induces defense responses to prevent pathogen colonization [44]. Since none of the AtPAOs are localized in the apoplast, it could be hypothesized that the protective roles attributed to apoplastic PAO in tobacco could be mediated by CuAOs in Arabidopsis. Further studies using knock-out mutants or *CuAO* overexpressor lines might help to verify this hypothesis. It is worth noting that *AtCuAO1* was strongly induced by SA, while *AtCuAO2* expression increased markedly in response to MeJA (Figure 5). SA and jasmonic acid induce resistance to biotrophic and necrotrophic pathogens, respectively [45]. Thus, the different response of *AtCuAO1* and *AtCuAO2* to these compounds, together with the different localization of the corresponding proteins (Figure 3), suggest a divergent evolution of CuAO family members leading to specificities in the response to biotrophic and necrotrophic pathogens. Indeed, in tobacco leaf apoplast, PA oxidation has been reported to have a beneficial effect in defence against biotrophic pathogens, while the opposite was observed for necrotrophic pathogens [35,46].

Overall, our results support that PA catabolism in Arabidopsis occurs in different cellular compartments mediated by different AOs (Figure 6). In the apoplast, Put and Spd are oxidized through a terminal process mediated by CuAOs (ATAO1, AtCuAO1), while in peroxisomes this pathway (catalysed by AtCuAO2 and AtCuAO3) is co-localized with the PAO-mediated back-conversion. In the cytosol, AtPAO1, and probably AtPAO5, catalyse the PA inter-conversion reactions [12]. Some of the putative AtCuAOs are annotated as cytosolic by TAIR, but no experimental data about their function and cell localization are available. Thus, the possibility of co-localization of both PA catabolic pathways in the cytosol cannot be discarded.

## Conclusions

Three Arabidopsis CuAOs involved in the terminal catabolism of Put and Spd, two major PAs present in plants, have been characterized. Our results indicate the occurrence of a differential localization of these CuAOs in the apoplast and peroxisomes, together with a possible functional specification of CuAOs against different types of stress. Importantly, our results support that back-conversion and terminal PA catabolism are co-localized in peroxisomes of Arabidopsis, thus suggesting that PA homeostasis is maintained by a tight coordination between both catabolic enzyme machineries (Figure 6). We propose a plausible and integrative model by which PA catabolism can be accomplished in Arabidopsis (Figure 6).

## Methods

### Sequence analysis and plasmid acquisition

Genomic database searches were performed using The Arabidopsis Information Resource (TAIR) database. Multiple sequence alignment of the amino acid sequences was done using the program ClustalW [47]. The plant expression vectors pYL436 [pC-TAPa, Gene Bank: AY737283] [27], pEarleyGate101 and pEarleyGate104 [48], as well as the plasmid containing the coding region of *CuAO1* fused to a TAP tag (DKLAT1G62810) were obtained from the Arabidopsis Biological Resource Centre (ABRC) (http://www.arabidopsis.org) (stocks CD3-679, CD3-683, CD3-686 and DKLAT1G62810, respectively). The peroxisomal marker, CFP-SKL [31] was also purchased by the ABRC (Stock CD3-977), while the extracellular marker Spg-DsRed [32] was kindly provided by Dr. MD Ludevid (Center for Research in Agricultural Genomics, Barcelona, Spain).

### Plant materials, growth conditions and treatments

*Arabidopsis thaliana* accession Columbia (Col-0) plants grown in soil or sterile seedlings were used in this work. For growth on soil, seeds were sown in pots containing a mixture of soil, perlite and vermiculite (1:1:1, v/v/v). To obtain sterile seedlings, surface sterilized seeds were sown in plates containing half-strength MS medium [49] supplemented with 1% (w/v) sucrose and solidified with 0.8% (w/v) plant agar (Duchefa Biochemie, Belgium). All seedlings and plants were stratified for 3 d at 4°C in the dark before being transferred to a growth chamber under 16 h /8 h light/dark cycles, at 100 mE m^–2^ s^–1^ of light intensity and a temperature of 22°C.

For treatment of seedlings with different compounds, 7-day-old seedlings, grown in MS-plates in a vertical position, were transferred to 6 well tissue culture clusters (10 seedlings/well) containing 8 ml of MS medium without agar and grown in the same conditions. After 10 days, the medium in the wells was removed and 8 ml of fresh medium, containing the different compounds to be tested: 500 μM SA, 100 μM MeJA, 20 μM ACC, 100 μM ABA or 1 μM flagellin 22, was added. Wounding was performed by thoroughly crushing the plantlets with forceps as described by [50].

### RNA isolation and quantitative RT–PCR analysis

RNA isolation and real-time PCR analyses were performed essentially as described by [51]. Total RNA from plant tissue was isolated using the TRIzol reagent (Invitrogen Life Technologies, Carlsbad, CA, USA) and subjected to DNase I RNase-free treatment (Invitrogen Life Technologies) to avoid amplification from genomic DNA. After checking the RNA in agarose gel, the samples were subjected to RT-PCR reactions using the Super-Script III first-strand synthesis system (Invitrogen Life Technologies). Real-time PCR assays were performed in quadruplicate with SYBR Green I Master (Roche Applied Science, Mannheim, Germany) using a Light Cycler 480 detection system (Roche). Specific primer sets were designed for *AtCuAO1*, 5’- AGCTGGCGACATTCTGAGAT-3’ (forward) and 5’-GTCCAGCATCATCCTCCCTA-3’ (reverse); *AtCuAO2,* 5’-GTCAAGATGGAACTCCCGC-3’ (forward) and 5’-TCGCCACATGATATCTCCAG-3’ (reverse), and *AtCuAO3,* 5’-GTAAGTTTGTGCCACTCCCCC-3’ (forward), 5’-GCCACTCGACAAAGTAACCCC-3’ (reverse). The amount of target mRNA was normalized by using *Actin2* as a housekeeping gene [52]. Experiments were repeated at least three times.

### Plasmid construction

The coding regions of *AtCuAO2* and *AtCuAO3* were amplified by RT-PCR from total RNA of *A. thaliana* leaves using the following gene-specific oligonucleotides: *AtCuAO2*-FWD (5’-CACCATGGCTCCACTTCAC-3’) and *AtCuAO2*-REV (5’-AAGATCGTTTTTAGGAGTGCAC-3’), *AtCuAO3*-FWD (5’-CACCATGGCCTCAGCTTCGAA-3’) and *AtCuAO3*-REV (5’-GCGAAGCTTTGAAAGTAAACCAGTCT-3’). The underlined regions in the forward primers facilitate the directional incorporation of the insert into the vector, and the reverse primers were designed to eliminate the stop codon. To maintain the stop codon of *AtCuAO3* cDNA, the *AtCuAO3*-REV-stop primer (5’-AAGCTTTGAAAGTAAACCAGTCTGA-3’) was used in combination with *AtCuAO3*-FWD. The PCR products were purified using the QIAquick gel extraction kit (Qiagen, CA, USA) and cloned into the pENTR/D-TOPO vector using Gateway technology according to the protocol provided by the manufacturer (Invitrogen Life Technologies). After being checked by sequencing, the inserts were transferred into the plant expression vectors pC-TAPa, pEarlyGate101 or pEarlyGate104. Cloning of the inserts into the pC-TAPa vector yielded the plasmids AtCuAO2-pC-TAP and AtCuAO3-pC-TAP. AtCuAO1-pC-TAP was obtained from the ABRC stock center (DKLAT1G62810). These plasmids, used for production and purification of the recombinant fusion proteins, contain the *AtCuAO1-3* coding sequences, fused at their C-terminus to a TAP tag, composed of two copies of the immunoglobulin-binding domain of protein A from *Staphylococcus aureus*, a human rhinovirus 3C protease cleavage site, a six histidine repeat and nine myc epitopes [27]. This is flanked by two copies of the cauliflower mosaic virus 35S promoter (2x35S) and a Nos terminator (NOSt) sequence at the 5’ and 3’ end, respectively (Figure 2). For protein localization studies, the amplified *AtCuAO2* and *AtCuAO3* cDNAs, without and with a stop codon, respectively, were transferred from the pENTR/D-TOPO vector to the C-terminal and N-terminal YFP binary fusion vectors pEarlyGate101 or pEarlyGate104, respectively (AtCuAO2-YFP, YFP-AtCuAO3). AtCuAO1-YFP was obtained by transferring the *AtCuAO1* coding region from AtCuAO1-pC-TAP to the pDONR221 vector, and from this to pEarlyGate101 using the Gateway recombination system. All vectors prepared were directly sequenced.

### Protein expression in *Nicotiana benthamiana* and purification

*Agrobacterium tumefaciens* strain EHA105 cells were transformed with the AtCuAO1-pC-TAP, AtCuAO2-pC-TAP or AtCuAO3-pC-TAP plasmids using the freeze-thaw method and PCR confirmed.

A single *Agrobacterium* colony containing the construct of interest was cultured in liquid YEB medium overnight at 28°C with appropriate antibiotics. The bacteria were pelleted at 3 000 *g* for 20 min, resuspended in infiltration media (10 mM MES pH 5.7, 10 mM MgCl_2_ and 200 μM acetosyringone), adjusted to an OD_600_ of 0.5 and incubated at room temperature for at least 3 h. *Agrobacterium* cultures transformed with the tobacco etch potyvirus helper component protein (HC-Pro) silencing suppressor [53] were resuspended in infiltration media to the same OD_600_. Equal volume of cultures containing the construct of interest and HC-Pro were mixed and infiltrated into 4-week-old *Nicotiana benthamiana* plants using a 1 ml needleless syringe. Three days after infiltration, the leaves were collected and stored at −80°C.

For protein extraction and purification, the procedure described by [27] was followed with some modifications. Collected leaves were ground in liquid nitrogen and homogenized with one volume of buffer containing: 100 mM Tris–HCl pH 7.5, 150 mM NaCl, 10% glycerol, 0.1% TritonX-100, 1 mM PMSF and 1x complete protease inhibitor cocktail (Sigma, St. Louis, MO, USA). Homogenates were filtered through four layers of cheesecloth and centrifuged at 14 000 g for 10 min at 4°C. Protein concentration in the supernatant was determined by the Bradford assay (Bio-Rad, Hercules, CA, USA). The total protein extracts were incubated with IgG Sepharose 6 Fast Flow beads (Amersham Biosciences, Uppsala, Sweden) for 2 h at 4°C with gentle rotation. After centrifugation at 150 *g* for 3 min at 4°C, the IgG beads were recovered and washed three times with 10 ml of washing buffer (extraction buffer plus 350 mM NaCl). Elution from the IgG beads was performed by incubation with 12 μl (50 units) of 3C protease (Precision protease; Amersham Biosciences) in 5 ml of washing buffer at 4°C with gentle rotation overnight. Supernatants were recovered after centrifugation at 150 *g* for 3 min at 4°C and stored. The IgG beads were washed with 5 ml of washing buffer and centrifuged again. Supernatants were recovered and, after pooling the eluates, proteins were concentrated using an Amicon Ultra-15 Centrifugal Filter (Millipore Corporation, Billerica, MA, USA) and separated on 8% SDS-PAGE gel. Protein bands were visualized by immunoblotting using the α-myc antibody.

### *In Vitro* activity assay

The CuAO activity of the recombinant fusion-proteins (AtCuAO1-MYC9-His6, AtCuAO2-MYC9-His6, AtCuAO3-MYC9-His6) was assayed by measuring the formation of H_2_O_2_ by fluorometry using the Amplex-Red hydrogen peroxide assay according to the manufacturer’s instructions (Invitrogen Life Technologies). Thus, the purified fusion protein (1 μg), or the same volume of elution buffer as a control, was incubated with 1 mM of each substrate, namely Put, Spd or Spm, 50 μM Amplex Red reagent, and 0.1 U ml^-1^ horseradish peroxidase in 100 mM Tris–HCl buffer (pH 8.0), at 37°C for 1 h. Fluorescence was measured at 540 nm excitation and 590 nm emission with a SpectraMax M3 Multi-Mode Microplate Reader (Molecular Devices, Sunnyvale, CA, USA), using black 96-well plates (Costar, Corning Incorporated, USA). Correction for background fluorescence was made by subtracting the values of reaction medium without the substrate. Amine oxidase-inhibited reactions were pre-treated with 0.5 mM AG or 30 μM 8 HQL for 5 min at 30°C and then subjected to the assay procedure described above. All experiments were repeated three times

### Protein localization analysis

*A. tumefaciens* strain EHA105 cells were transformed with the plasmids containing the fusion proteins AtCuAO1-YFP, AtCuAO2-YFP and YFP-AtCuAO3 and infiltrated into 3–4 week old *N. benthamiana* plants as described above.

For colocalization experiments, suspensions of *A. tumefaciens* harbouring the AtCuAO1-YFP expression construct were mixed with *A. tumefaciens* cultures harbouring constructs for expression of the red fluorescent protein (DsRed)-extracellular marker (Spg-DsRed) [32], while *A. tumefaciens* harbouring the AtCuAO2-YFP and YFP-AtCuAO3 expression constructs were mixed with bacteria cultures containing constructs for expression of the cyan fluorescent protein (CFP)-peroxisome marker (CFP-SKL). In both cases, *A. tumefaciens* cultures containing HC-Pro were added to the mixture in a ratio of 1:1:2 (v:v:v) of fusion protein:marker:HC-Pro to suppress silencing. Agroinfiltrated leaves were examined after 3–4 days by confocal laser-scanning microscopy using an Olympus FV1000 microscope (Olympus, Japan). We used the 515-, 405- and 595-nm laser to excite YFP, CFP and DsRed, respectively. Fluorescence was detected using an emission filter of a 530- to 630-nm band pass for YFP and DsRed and a 460- to 500-nm band pass for CFP. All images were acquired from single optical sections. Images were merged using the FV1000 Viewer software Ver.03.1 (Olympus, Japan).

## Abbreviations

ABA: Abscisic acid; ACC: 1-aminocyclopropane-1-carboxylic acid; ADH: Aldehyde deshydrogenase; AG: Aminoguanidine; AO: Amine oxidase; APAL: 3-aminopropanal; BADH: Betaine aldehyde deshydrogenase; CuAO: Cooper-containing amine oxidase; GABA: γ-aminobutiric acid; HQL: Hydroxyquinoline; MeJA: Methyl-jasmonate; PAO: Polyamine oxidase; PTS: Peroxisome targeting sequence; Put: Putrescine; SA: Salicylic acid; Spd: Spermidine; Spm: Spermine; TAP: Tandem affinity purification.

## Competing interests

The authors declare that they have not competing interests.

## Authors’ contributions

JP-P carried out the molecular genetic studies and the activity and localization assays, participated in the production of the recombinant proteins and in the design of the study, and helped to draft the manuscript. MG carried out the purification of the recombinant proteins. AFT participated in the design of the study and in the drafting of the manuscript. TA conceived of the study, participated in its design, performed the sequence alignment and drafted the manuscript. All the authors read and approved the final manuscript.

## References

[B1] Bouchereau A Aziz A Larher F Martin-Tanguy J Polyamines and environmental challenges: recent development Plant Sci 1999 140 103 125 10.1016/S0168-9452(98)00218-0

[B2] Walters DR Polyamines and plant disease Phytochem 2003 64 97 107 10.1016/S0031-9422(03)00329-7 12946408

[B3] Kusano T Berberich T Tateda C Takahashi Y Polyamines: essential factors for growth and survival Planta 2008 228 367 381 10.1007/s00425-008-0772-7 18594857

[B4] Alcazar R Altabella T Marco F Bortolotti C Reymond M Koncz C Carrasco P Tiburcio AF Polyamines: molecules with regulatory functions in plant abiotic stress tolerance Planta 2010 231 1237 1249 10.1007/s00425-010-1130-0 20221631

[B5] Cohen SS A guide to the polyamines 1988 New York: Oxford University Press

[B6] Cona A Rea G Angelini R Federico R Tavladoraki P Functions of amine oxidases in plant development and defence Trends Plant Sci 2006 11 80 88 16406305 10.1016/j.tplants.2005.12.009

[B7] Angelini R Cona A Federico R Fincato P Tavladoraki P Tisi A Plant amine oxidases “on the move”: An update Plant Physiol Biochem 2010 48 560 564 10.1016/j.plaphy.2010.02.001 20219383

[B8] Tavladoraki P Rossi MN Saccuti G Perez-Amador MA Polticelli F Angelini R Federico R Heterologous expression and biochemical characterization of a polyamine oxidase from arabidopsis involved in polyamine back conversion Plant Physiol 2006 141 1519 1532 10.1104/pp.106.080911 16778015 PMC1533960

[B9] Moschou PN Sanmartin M Andriopoulou AH Rojo E Sanchez-Serrano JJ Roubelakis-Angelakis KA Bridging the gap between plant and mammalian polyamine catabolism: A novel peroxisomal polyamine oxidase responsible for a full back-conversion pathway in Arabidopsis Plant Physiol 2008 147 1845 1857 10.1104/pp.108.123802 18583528 PMC2492618

[B10] Kamada-Nobusada T Hayashi M Fukazawa M Sakakibara H Nishimura M A putative peroxisomal polyamine oxidase, AtPAO4, is involved in polyamine catabolism in *Arabidopsis thaliana* Plant Cell Physiol 2008 49 1272 1282 10.1093/pcp/pcn114 18703589

[B11] Takahashi Y Cong R Sagor GH Niitsu M Berberich T Kusano T Characterization of five polyamine oxidase isoforms in *Arabidopsis thaliana* Plant Cell Rep 2010 29 955 965 10.1007/s00299-010-0881-1 20532512

[B12] Fincato P Moschou PN Spedaletti V Tavazza R Angelini R Federico R Roubelakis-Angelakis KA Tavladoraki P Functional diversity inside the Arabidopsis polyamine oxidase gene family J Exp Bot 2011 62 1155 1168 10.1093/jxb/erq341 21081665

[B13] Seiler N Catabolism of polyamines Amino Acids 2004 26 217 233 15221502 10.1007/s00726-004-0070-z

[B14] Pietrangeli P Federico R Mondovì B Morpurgo L Substrate specificity of copper-containing plant amine oxidases J Inorg Biochem 2007 101 997 1004 10.1016/j.jinorgbio.2007.03.014 17521737

[B15] Medda R Padiglia A Floris G Plant copper-amine oxidases Phytochem 1995 39 1 9 10.1016/0031-9422(94)00756-J 10403363

[B16] Tipping AJ McPherson MJ Cloning and molecular analysis of the pea seedling copper amine oxidase J Biol Chem 1995 270 16939 16946 10.1074/jbc.270.28.16939 7622512

[B17] Møller SG McPherson MJ Developmental expression and biochemical analysis of the Arabidopsis atao1 gene encoding an H2O2-generating diamine oxidase Plant J 1998 13 781 791 10.1046/j.1365-313X.1998.00080.x 9681017

[B18] Rea G de Pinto MC Tavazza R Biondi S Gobbi V Ferrante P De Gara L Federico R Angelini R Tavladoraki P Ectopic expression of maize polyamine oxidase and pea copper amine oxidase in the cell wall of tobacco plants Plant Physiol 2004 134 1414 1426 10.1104/pp.103.036764 15064377 PMC419818

[B19] Federico R Angelini R Slocum RD, Flores HE Polyamine catabolism in plants Biochemistry and Physiology of Polyamines in Plants 1991 Boca Raton: CRC Press 41 56

[B20] Zimmermann P Hirsch-Hoffmann M Hennig L Gruissem W GENEVESTIGATOR. arabidopsis microarray database and analysis toolbox Plant Physiol 2004 136 2621 2632 10.1104/pp.104.046367 15375207 PMC523327

[B21] Wimalasekera R Villar C Begum T Scherer GF COPPER AMINE OXIDASE1 (CuAO1) of *Arabidopsis thaliana* contributes to abscisic acid- and polyamine-induced nitric oxide biosynthesis and abscisic acid signal transduction Mol Plant 2011 4 663 678 10.1093/mp/ssr023 21471330

[B22] Janes SM Palcic MM Scaman CH Smith AJ Brown DE Dooley DM Mure M Klinman JP Identification of topaquinone and its consensus sequence in copper amine oxidases Biochem 1992 31 12147 12154 10.1021/bi00163a025 1457410

[B23] Mu D Janes SM Smith AJ Brown DE Dooley DM Klinman JP Tyrosine codon corresponds to topa quinone at the active site of copper amine oxidases J Biol Chem 1992 267 7979 7982 1569055

[B24] Parsons MR Convery MA Wilmot CM Yadav KDS Blakeley V Corner AS Phillips SEV McPherson MJ Knowles PF Crystal structure of a quinoenzyme: copper amine oxidase of *Escherichia coli* at 2 å resolution Structure 1995 3 1171 1184 10.1016/S0969-2126(01)00253-2 8591028

[B25] Kumar V Dooley DM Freeman HC Guss JM Harvey I McGuirl MA Wilce MCJ Zubak VM Crystal structure of a eukaryotic (pea seedling) copper-containing amine oxidase at 2.2 å resolution Structure 1996 4 943 955 10.1016/S0969-2126(96)00101-3 8805580

[B26] Wilmot CM Murray JM Alton G Parsons MR Convery MA Blakeley V Corner AS Palcic MM Knowles PF McPherson MJ Phillips SE Catalytic mechanism of the quinoenzyme amine oxidase from *Escherichia coli*: exploring the reductive half-reaction Biochem 1997 36 1608 1620 10.1021/bi962205j 9048544

[B27] Rubio V Shen Y Saijo Y Liu Y Gusmaroli G Dinesh-Kumar SP Deng XW An alternative tandem affinity purification strategy applied to Arabidopsis protein complex isolation Plant J 2005 41 767 778 10.1111/j.1365-313X.2004.02328.x 15703063

[B28] Eubel H Meyer EH Taylor NL Bussell JD O'Toole N Heazlewood JL Castleden I Small ID Smith SM Millar AH Novel proteins, putative membrane transporters, and an integrated metabolic network are revealed by quantitative proteomic analysis of Arabidopsis cell culture peroxisomes Plant Physiol 2008 148 1809 1829 10.1104/pp.108.129999 18931141 PMC2593673

[B29] Reumann S Quan S Aung K Yang P Manandhar-Shrestha K Holbrook D Linka N Switzenberg R Wilkerson CG Weber APM Olsen LJ Hu J In-Depth proteome analysis of arabidopsis leaf peroxisomes combined with in vivo subcellular targeting cerification indicates novel metabolic and regulatory functions of peroxisomes Plant Physiol 2009 150 125 143 10.1104/pp.109.137703 19329564 PMC2675712

[B30] Heazlewood JL Verboom RE Tonti-Filippini J Small I Millar AH SUBA: the Arabidopsis Subcellular Database Nucl Acids Res 2007 35 Database issue D213 D218 17071959 10.1093/nar/gkl863 PMC1635339

[B31] Nelson BK Cai X Nebenfuhr A A multicolored set of in vivo organelle markers for co-localization studies in Arabidopsis and other plants Plant J 2007 51 1126 1136 10.1111/j.1365-313X.2007.03212.x 17666025

[B32] Torrent M Llop-Tous I Ludevid MD Protein body induction: a new tool to produce and recover recombinant proteins in plants Meth Mol Biol 2009 483 193 208 10.1007/978-1-59745-407-0_11 19183900

[B33] Agostinelli E Arancia G Vedova LD Belli F Marra M Salvi M Toninello A The biological functions of polyamine oxidation products by amine oxidases: Perspectives of clinical applications Amino Acids 2004 27 347 358 10.1007/s00726-004-0114-4 15592759

[B34] Moschou PN Paschalidis KA Roubelakis-Angelakis KA Plant polyamine catabolism. The state of the art Plant Signal Behav 2008 3 1061 1066 10.4161/psb.3.12.7172 19513239 PMC2634460

[B35] Tavladoraki P Cona A Federico R Tempera G Viceconte N Saccoccio S Battaglia V Toninello A Agostinelli E Polyamine catabolism: target for antiproliferative therapies in animals and stress tolerance strategies in plants Amino Acids 2012 42 411 416 10.1007/s00726-011-1012-1 21874532

[B36] Sebela M Brauner F Radova A Jacobsen S Havlis J Galuszka P Pec P Characterisation of a homogeneous plant aminoaldehyde dehydrogenase Biochim Biophys Acta 2000 1480 329 341 10.1016/S0167-4838(00)00086-8 11004571

[B37] Missihoun T Schmitz J Klug R Kirch H-H Bartels D Betaine aldehyde dehydrogenase genes from Arabidopsis with different sub-cellular localization affect stress responses Planta 2011 233 369 382 10.1007/s00425-010-1297-4 21053011

[B38] Paschalidis KA Roubelakis-Angelakis KA Sites and regulation of polyamine catabolism in the tobacco plant. Correlations with cell division/expansion, cell cycle progression, and vascular development Plant Physiol 2005 138 2174 2184 10.1104/pp.105.063941 16040649 PMC1183405

[B39] Tisi A Angelini R Cona A Wound healing in plants: Cooperation of copper amine oxidase and flavin-containing polyamine oxidase Plant Signal Behav 2008 3 204 206 10.4161/psb.3.3.5372 19704660 PMC2634118

[B40] Rea G Metoui O Infantino A Federico R Angelini R Copper amine oxidase expression in defense responses to wounding and *Ascochyta rabiei* invasion Plant Physiol 2002 128 865 875 10.1104/pp.010646 11891243 PMC152200

[B41] Petrivalsky M Brauner F Luhova L Gagneul D Sebela M Aminoaldehyde dehydrogenase activity during wound healing of mechanically injured pea seedlings J Plant Physiol 2007 164 1410 1418 10.1016/j.jplph.2007.01.018 17728013

[B42] Yoda H Hiroi Y Sano H Polyamine oxidase is one of the key elements for oxidative burst to induce programmed cell death in tobacco cultured cells Plant Physiol 2006 142 193 206 10.1104/pp.106.080515 16844838 PMC1557616

[B43] Moschou PN Paschalidis KA Delis ID Andriopoulou AH Lagiotis GD Yakoumakis DI Roubelakis-Angelakis KA Spermidine exodus and oxidation in the apoplast induced by abiotic stress is responsible for H_2_O_2_ signatures that direct tolerance responses in tobacco Plant Cell 2008 20 1708 1724 10.1105/tpc.108.059733 18577660 PMC2483379

[B44] Moschou PN Sarris PF Skandalis N Andriopoulou AH Paschalidis KA Panopoulos NJ Roubelakis-Angelakis KA Engineered polyamine catabolism preinduces tolerance of tobacco to bacteria and oomycetes Plant Physiol 2009 149 1970 1981 10.1104/pp.108.134932 19218362 PMC2663742

[B45] Thaler JS Humphrey PT Whiteman NK Evolution of jasmonate and salicylate signal crosstalk Trends Plant Sci 2012 17 5 260 270 10.1016/j.tplants.2012.02.010 22498450

[B46] Marina M Maiale SJ Rossi FR Romero MF Rivas EI Garriz A Ruiz OA Pieckenstain FL Apoplastic polyamine oxidation plays different roles in local responses of tobacco to infection by the necrotrophic fungus Sclerotinia sclerotiorum and the biotrophic bacterium Pseudomonas viridiflava Plant Physiol 2008 147 2164 2178 10.1104/pp.108.122614 18583531 PMC2492638

[B47] Thompson JD Higgins DG Gibson TJ Improved sensitivity of profile searches through the use of sequence weights and gap excision Computer applications in the biosciences: CABIOS 1994 10 19 29 8193951 10.1093/bioinformatics/10.1.19

[B48] Earley KW Haag JR Pontes O Opper K Juehne T Song K Pikaard CS Gateway-compatible vectors for plant functional genomics and proteomics Plant J 2006 45 616 629 10.1111/j.1365-313X.2005.02617.x 16441352

[B49] Murashige T Skoog F A Revised medium for rapid growth and bio assays with tobacco tissue cultures Physiol Plant 1962 15 473 497 10.1111/j.1399-3054.1962.tb08052.x

[B50] Rojo E Titarenko E León J Berger S Vancanneyt G Sánchez-Serrano JJ Reversible protein phosphorylation regulates jasmonic acid-dependent and -independent wound signal transduction pathways in *Arabidopsis thaliana* Plant J 1998 13 153 165 10.1046/j.1365-313X.1998.00020.x 9680973

[B51] Cuevas JC Lopez-Cobollo R Alcazar R Zarza X Koncz C Altabella T Salinas J Tiburcio AF Ferrando A Putrescine is involved in Arabidopsis freezing tolerance and cold acclimation by regulating abscisic acid levels in response to low temperature Plant Phys 2008 148 1094 1105 10.1104/pp.108.122945 PMC2556839 18701673

[B52] Alcázar R García-Martinez JL Cuevas JC Tiburcio AF Altabella T Overexpression of *ADC2* in Arabidopsis induces dwarfism and late-flowering through GA deficiency Plant J 2005 43 425 436 10.1111/j.1365-313X.2005.02465.x 16045477

[B53] Goytia E Fernandez-Calvino L Martinez-Garcia B Lopez-Abella D Lopez-Moya JJ Production of plum pox virus HC-Pro functionally active for aphid transmission in a transient-expression system J Gen Vir 2006 87 3413 3423 10.1099/vir.0.82301-0 17030878

